# Approaches in Hydroxytyrosol Supplementation on Epithelial—Mesenchymal Transition in TGFβ1-Induced Human Respiratory Epithelial Cells

**DOI:** 10.3390/ijms24043974

**Published:** 2023-02-16

**Authors:** Rabiatul Adawiyah Razali, Muhammad Dain Yazid, Aminuddin Saim, Ruszymah Bt Hj Idrus, Yogeswaran Lokanathan

**Affiliations:** 1 Centre for Tissue Engineering and Regenerative Medicine, Faculty of Medicine, Universiti Kebangsaan Malaysia, Cheras, Kuala Lumpur 56000, Malaysia; 2 Graduate School of Medicine, KPJ Healthcare University College, Kota Seriemas, Nilai 71800, Malaysia; 3 Department of Physiology, Faculty of Medicine, Universiti Kebangsaan Malaysia, Cheras, Kuala Lumpur 56000, Malaysia

**Keywords:** olive, EMT, fibrosis, inflammation, rhinosinusitis

## Abstract

Hydroxytyrosol (HT) is an olive polyphenol with anti-inflammatory and antioxidant properties. This study aimed to investigate the effect of HT treatment on epithelial–mesenchymal transition (EMT) in primary human respiratory epithelial cells (RECs) isolated from human nasal turbinate. HT dose–response study and growth kinetic study on RECs was performed. Several approaches on HT treatment and TGFβ1 induction with varying durations and methods was studied. RECs morphology and migration ability were evaluated. Vimentin and E-cadherin immunofluorescence staining and Western blotting [E-cadherin, vimentin, SNAIL/SLUG, AKT, phosphorylated (p)AKT, SMAD2/3 and pSMAD2/3] were performed after 72-h treatment. In silico analysis (molecular docking) of HT was performed to evaluate the potential of HT to bind with the TGFβ receptor. The viability of the HT-treated RECs was concentration-dependent, where the median effective concentration (EC_50_) was 19.04 μg/mL. Testing of the effects of 1 and 10 µg/mL HT revealed that HT suppressed expression of the protein markers vimentin and SNAIL/SLUG while preserving E-cadherin protein expression. Supplementation with HT protected against SMAD and AKT pathway activation in the TGFβ1-induced RECs. Furthermore, HT demonstrated the potential to bind with ALK5 (a TGFβ receptor component) in comparison to oleuropein. TGFβ1-induced EMT in RECs and HT exerted a positive effect in modulating the effects of EMT.

## 1. Introduction

Approximately 2.7–8% of Asians and 5–15% of the worldwide population are affected by chronic rhinosinusitis (CRS). Rhinosinusitis is characterized by paranasal sinus inflammation, while the symptoms for CRS include nasal blockage, congestion, and mucus discharge. Generally, these symptoms could be alleviated by nasal irrigation, antihistamines, antibiotics, and intranasal corticosteroids [1,2]. However, prolonged inflammation in rhinosinusitis that leads to CRS can also be accompanied by nasal polyps. Given its debilitating nature, CRS generally can affect the patient’s quality of life and productivity.

The normal epithelial cell layer that covers the respiratory tract consists of ciliated, goblet, and basal cell [3]. Prolonged invasion of foreign substances and pathogens damage and injure the epithelial layer. During injury, basal cells downregulate their epithelial protein expression (E-cadherin and cytokeratin) and lose polarity. The cells will highly express mesenchymal proteins (vimentin and αSMA) and regain the ability to migrate to the injury region, proliferate, and eventually differentiate back to ciliated epithelial cells and goblet cells [4,5]. The tissue remodeling and ability of the polarized epithelial cells to gain the mesenchymal phenotype, such as migration, and then differentiate back to epithelial cells, are termed epithelial–mesenchymal transition (EMT) and mesenchymal–epithelial transition, respectively.

EMT activation involves several molecular pathways, such as the TGFβ pathway, Notch pathway, or pathways activated through growth factor binding to tyrosine kinase receptors (RTK), such as hepatocyte growth factor (HGF) and fibroblast growth factor (FGF) [6]. Among these pathways, the TGFβ pathway is the most important pathway in EMT activation. The interaction of the TGFβ growth factor with its receptors (TβRI/Alk5) and TβRII will activate the SMAD-dependent EMT pathway through phosphorylation of SMAD2/3 [7].

These molecular mechanisms are important for wound healing and normal remodeling processes. However, prolonged injury or chronic diseases will lead to modification and changes in tissue and organ components and architecture through mechanisms such as EMT, which will eventually lead to a pathological event. Evidence of tissue remodeling such as epithelial damage and basement thickening is present in patients with CRS [8]. It has been demonstrated that EMT and CRS are related [3,9,10]. TGFβ1 increased as wound repair and remodeling took place in patients with CRS [11]. Nasal polyps, which are the result of tissue remodeling, might also be present in patients with CRS [12]. EMT mechanisms have also been reported in both nasal polyps and CRS events. [3,12,13,14]. Tissue remodeling is largely irreversible and can cause obstructions and breathing difficulties. These issues will eventually require surgery to improve ventilation, such as functional endoscopic sinus surgery.

Therefore, the modulation of the EMT mechanism should be investigated to manage diseases. Recent studies have reported the ability of natural products to modulate the EMT pathway [15,16,17]. Commonly known as olive, *Olea europaea (OE)* is a promising natural product with proven health benefits due to its phenolic and flavonoid content [18]. Olive oil is the primary source of added fat in the Mediterranean diet, which has been associated with health benefits such as reducing neurodegenerative diseases, improving bone health, and demonstrating anti-cancer properties [19]. Several studies have reported the ability of OE phenolic compounds in EMT modulation in fibrosis and breast cancer cells. However, there have been few scientific studies on the ability of olive fruits to treat airway diseases. Interestingly, olive leaves have been used to treat asthma traditionally [20]. Other than that, OE and its active compounds can also act as antioxidant agents on lung epithelial cells and reduce inflammation in lung tissue [21,22].

Approximately 1–2% of the OE phenolic compound content comprises of oleuropein, tyrosol, and hydroxytyrosol (HT). The hydrolysis of oleuropein, which typically occurs during olive maturation, oil storage, and preparation of table olives, yields HT [23]. Besides being affected by the process, the amount of HT obtained also depends on the quality of the olive oil and the types of olive. Black olives have a total phenolic content of 16.40 g per kg of dry weight, of which 5.78 g per kg is HT itself. However, the content of HT in green olives is lower at 4.48 g kg [24].

HT is also claimed to be beneficial to health by acting as a cardiovascular health protector, antioxidant, ROS scavenger, anti-inflammatory, anti-cancer, antimicrobial, neuroprotective, prevent osteoporosis and provide skin and eye health benefits [18,23,25,26]. Besides that, HT is also a natural antioxidant with the strongest potential compared to all olive polyphenols. In addition, it also has twice the antioxidant power of coenzyme Q10 [26]

Oleuropein, tyrosol and HT been shown to modulate the EMT signaling pathway through ligand binding via the ERK, AKT or WNT pathway [27]. However, there were only several studies that have highlighted the beneficial effect of HT on the modulation of EMT signaling pathway [28,29,30]. Therefore, we investigated the effect of HT supplementation on TGFβ1-induced human respiratory epithelial cells (RECs).

## 2. Results

### 2.1. Dose-Dependent Effect of HT on RECs

**Figure 1:**
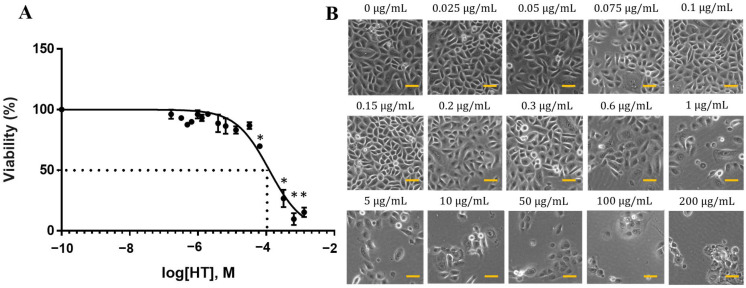
Dose-dependent effect of HT on RECs. RECs were treated with HT for 24 h, and cell viability was analyzed via MTT assay. (**A**) MTT assay of HT cytotoxicity. * *p* < 0.05: significantly different from control (0 μg/mL) (Supplementary Material Table S1). (**B**) Morphology of RECs treated with HT for 24 h. Scale bar represent 100 µm.

We examined the dose-dependent effects of HT on normal RECs. The cells treated with up to 5 μg/mL HT maintained their viability after 24 h. However, concentration-dependent inhibition of cell proliferation was observed beginning from 10 μg/mL HT, with a median effective concentration (EC_50_) of 19.04 μg/mL (123.5 μM) (Figure 1A). The REC viability percentage began to decrease following treatment with 0.6 μg/mL HT (Figure 1B). The concentrations around EC_50_ (0.05, 0.1, 0.2, 0.5, 1, 15, 30, and 50 μg/mL) were selected for the subsequent experiment.

### 2.2. Long-Term Effect of HT on REC Growth

**Figure 2:**
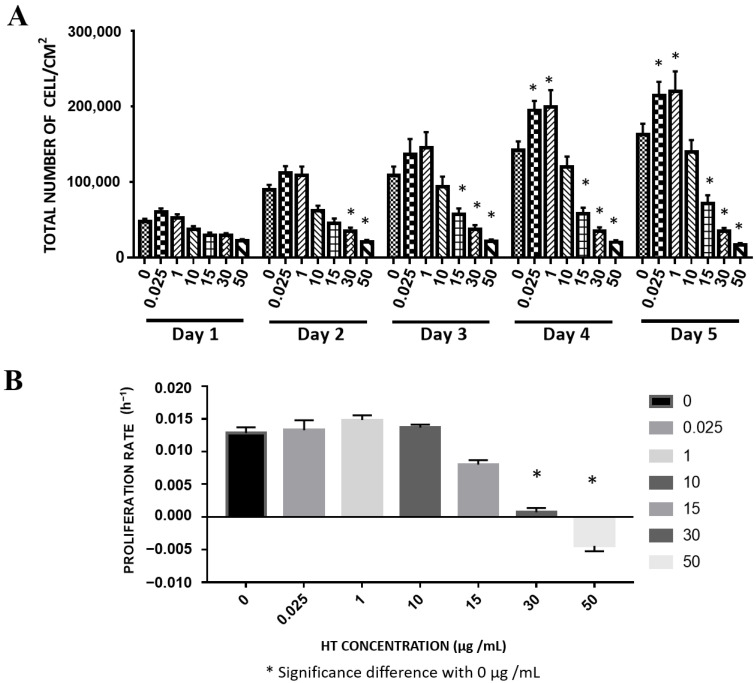
Long-term effect of HT supplementation on RECs. (**A**) Total number of cells attached vs. HT concentration. (**B**). Proliferation rate. * *p* < 0.05: significantly different from control (Supplementary Material Table S2).

We supplemented RECs with HT for up to 5 days to study the effect of long-term HT exposure on the cells. The cell number increased from day 1 to day 5 following treatment with low HT concentrations (0.025, 1, 10 μg/mL). This increasing trend was similar to the increasing trend of the total cell number in the untreated REC group. However, starting from day 4, the total cell numbers of RECs treated with 0.025 and 1 μg/mL HT were significantly higher than that of the control, while the total REC number of the 10 μg/mL HT group was not significantly different from that of the control. The 15, 30, and 50 μg/mL HT groups exhibited no significant increase in total cell number (Figure 2A). Meanwhile, RECs treated with 0.025, 1, and 10 μg/mL HT did not have a significantly different proliferation rate compared with the control. The proliferation rates of the RECs treated with 30 and 50 μg/mL HT were significantly lower than that of the control, thereby suggesting that the cells had stopped proliferating (Figure 2B).

### 2.3. HT Modulates EMT Marker Expression

**Figure 3:**
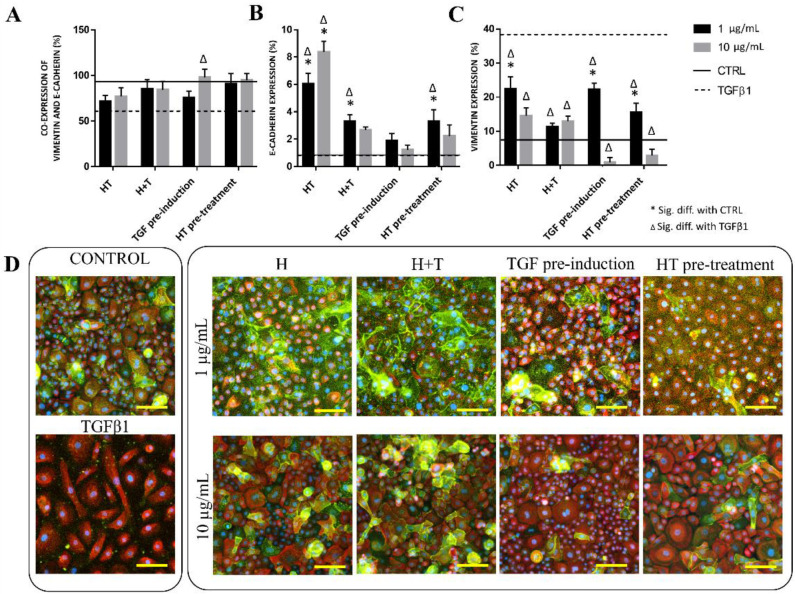
Protein expression levels of TGFβ1- and HT-treated RECs. (**A**) E-cadherin and vimentin co-expression levels (%) vs. control. (**B**) E-cadherin expression (%) vs. control. (**C**) Vimentin expression (%) vs. control. * *p* < 0.05: significantly different from control. # *p* < 0.05: significantly different from Group T (Supplementary Material Table S3). (**D**) Immunostaining of vimentin and E-cadherin expression levels at 72 h. Green cytoplasm indicates E-cadherin expression level, red indicates vimentin, and blue is DAPI-stained nuclei. Scale bar represent 100 µm.

We evaluated the protein expression levels of the RECs to examine the effect of HT on EMT events. Figure 3 depicts the E-cadherin and vimentin expression levels in each treatment group after 72 h. The E-cadherin expression levels in the 1 μg/mL H, 1 μg/mL H+T, 1 μg/mL HT pre-treatment, and 10 μg/mL H groups were significantly higher than those in the untreated and TGFβ1-induced RECs. All HT-treated groups had significantly lower vimentin expression than the TGFβ1-treated RECs, with 10 μg/mL TGF pre-induction leading to the lowest vimentin expression. At 72 h, more than 50% of the cells co-expressed E-cadherin and vimentin. However, the expression difference between the treatment groups was not significant except for the 10 μg/mL TGF pre-induction group (Figure 3).

### 2.4. HT Maintains REC Morphology

**Figure 4:**
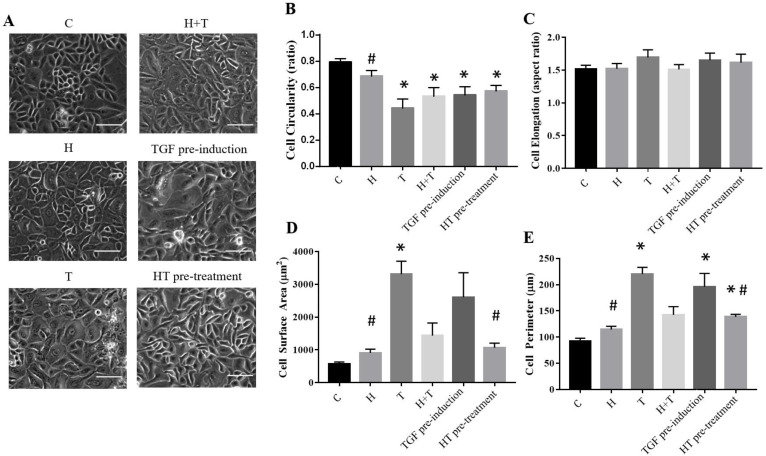
(**A**) REC morphology following culture in the six conditions. (**B**) Cell circularity. (**C**) Cell elongation. (**D**) Cell surface area. (**E**) Cell perimeter. * *p* < 0.05: significantly different from control. # *p* < 0.05: significantly different from Group T (Supplementary Material Table S4). Scale bar represent 100 µm.

The HT concentration of 10 μg/mL was used for subsequent experiments. The morphological changes of all tested groups were evaluated through cell circularity, elongation, cell surface, and cell perimeter analyses (Figure 4). The control RECs had a circularity value of 0.792 ± 0.029, which did not differ significantly from that of the HT-treated RECs (0.686 ± 0.045). This indicated that the control RECs and HT-treated cells were round and not fibroblastic. However, the TGFβ1-induced RECs were significantly different, where they were not circular and were elongated (0.443 ± 0.298). Group H+T demonstrated a similar circularity value to Group T, albeit the increased circularity values (0.532 ± 0.068, 0.5449 ± 0.06, and 0.573 ± 0.04) indicated that the RECs were able to maintain their polygonal shape even after being cultured with TGFβ1 (Figure 4). These results were in line with the cell elongation analysis, where the RECs induced by TGFβ1 (1.693 ± 0.1173) and TGF pre-induction (1.652 ± 0.1078) were more elongated than the RECs in the other groups (C: 1.512 ± 0.06174; H: 1.52 ± 0.08025; H+T: 1.51 ± 0.07461, HT pre-treatment: 1.612 ± 0.13). Additionally, the surface area and perimeter value of the cells induced with TGFβ1 (3311 ± 394 μm^2^) and TGF pre-induction (2605 ± 750.3 μm^2^) were higher compared to that of the other groups, indicating that the cell size was increased as compared to the control RECs (569.5 ± 63.16 μm^2^). HT supplementation maintained the cell surface area and perimeter values in Groups HT (907.5 ± 115.2 μm^2^), H+T (1440 ± 381.5 μm^2^), and HT pre-treatment (1065 ± 138.8 μm^2^).

### 2.5. HT Impedes Migration in TGFβ1-Induced RECs

**Figure 5:**
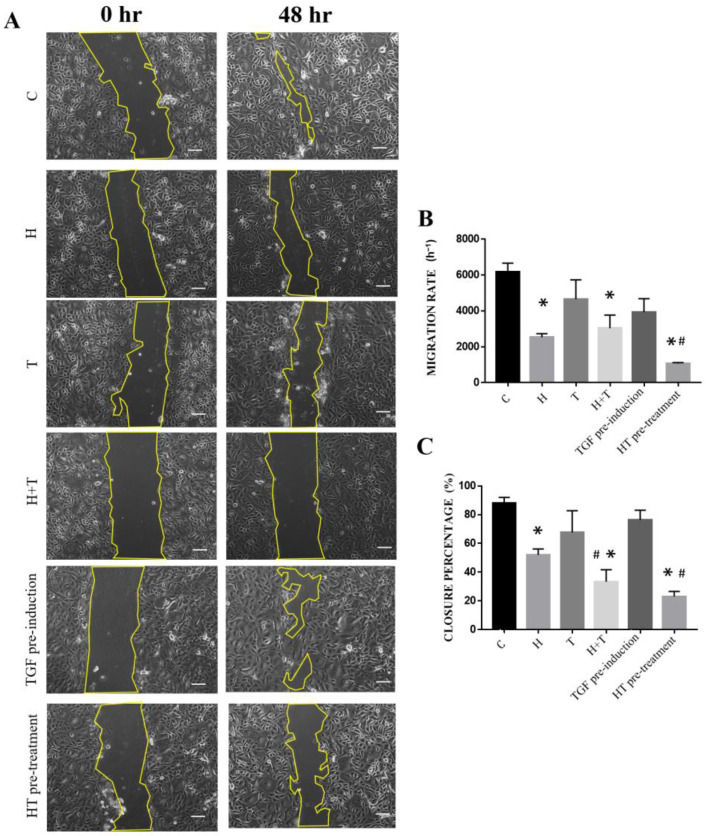
(**A**) Migration of RECs that had been cultured in six conditions. (**B**) Cell migration rate. (**C**) Scratch closure percentage. * *p* < 0.05: significantly different from control. # *p* < 0.05: significantly different from Group T (Supplementary Material Table S5). Scale bar represent 100 µm.

The migration rate of uninduced and untreated RECs was higher than that of the other groups (7115 ± 357.5 h^−1^). Group HT pre-treatment had the lowest migration rate (954.6 ± 121.8 h^−1^) compared to the other treatment groups. After 48 h, the scratch closure percentage of Groups H, H+T, and HT pre-treatment had a lower scratch closure rate (52.07 ± 4.06%; 33.39 ± 8.30%; 23.05 ± 3.53%, respectively) compared to the control RECs (87.9 ± 4.12%). Groups T and TGF pre-induction had higher scratch closure rates than the control RECs (Figure 5).

### 2.6. HT Attenuates pAKT and pSMAD2/3 Expression

**Figure 6:**
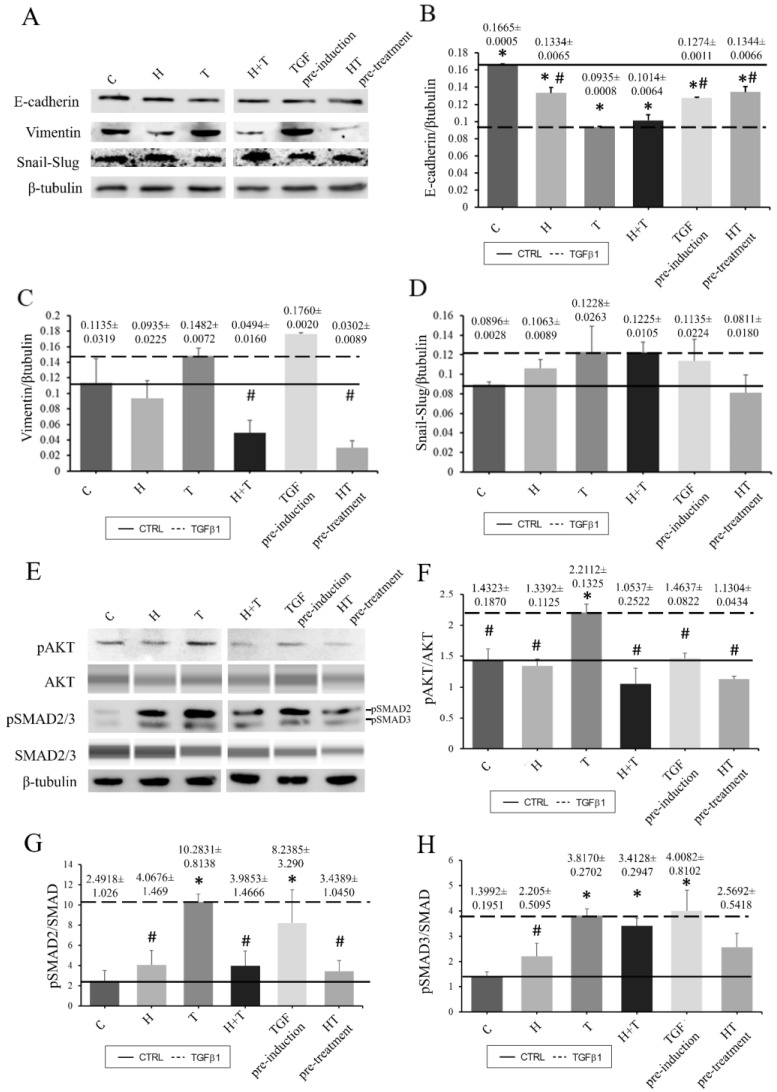
Densitometry analysis of tested protein markers to observe the effects of HT and TGFβ1 on RECs. All Western blots were performed using a conventional Western blot system except for AKT and SMAD2/3, which were analyzed with the Jess system (**A**–**H**). E-cadherin (**B**), Vimentin (**C**), Snail-Slug (**D**), pAKT (**F**), pSMAD2 (**G**) and pSMAD3 (**H**). Solid line: Untreated REC. Dashed line: TGFβ1-induced REC. * *p* < 0.05: significantly different from control. # *p* < 0.05: significantly different from Group T (Supplementary Material Table S6).

RECs without TGFβ1 induction or HT treatment demonstrated higher E-cadherin expression compared to the other groups. The TGFβ1-induced RECs had lower E-cadherin expression. However, culturing the RECs with HT prevented the effect of TGFβ1 from further repressing E-cadherin expression. The expression of vimentin as a mesenchymal marker was also examined. The control RECs expressed vimentin, but their expression rate was lower than that of the TGFβ1 group, while vimentin expression was highest in Group TGF pre-induction. HT treatment caused decreased vimentin expression, which was also observed in Groups H+T and HT pre-treatment, where the addition of HT was accompanied by decreased vimentin expression as compared to the control group (Figure 6). In addition to E-cadherin and vimentin, SNAIL/SLUG expression was also observed. HT and TGFβ1 caused increased SNAIL/SLUG expression. However, Group HT pre-treatment had lower SNAIL/SLUG expression than the TGFβ1-induced group (Figure 6). Furthermore, the effect of HT on AKT and SMAD2/3 phosphorylation was studied. TGFβ1 increased the phosphorylation activity on the AKT marker protein in normal RECs while the addition of HT reduced AKT phosphorylation activity. Among the three treatment groups (H+T, TGF pre-induction, HT pre-treatment), RECs in group TGF pre-induction had high AKT phosphorylation activity even when treated with HT (Figure 6). A similar reduction pattern of SMAD2/3 marker protein phosphorylation activity was observed for the TGFβ1-induced RECs when treated with HT (Figure 6).

### 2.7. Molecular Docking of HT Acetate, HT, Tyrosol and Oleuropein

**Figure 7:**
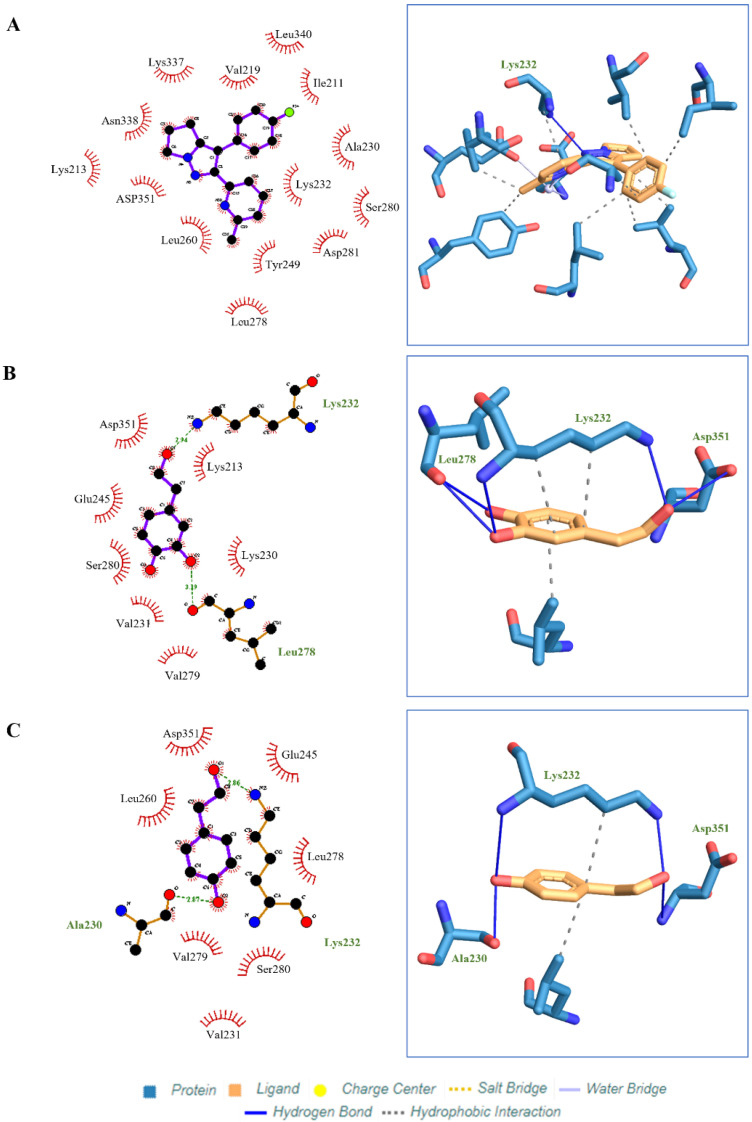

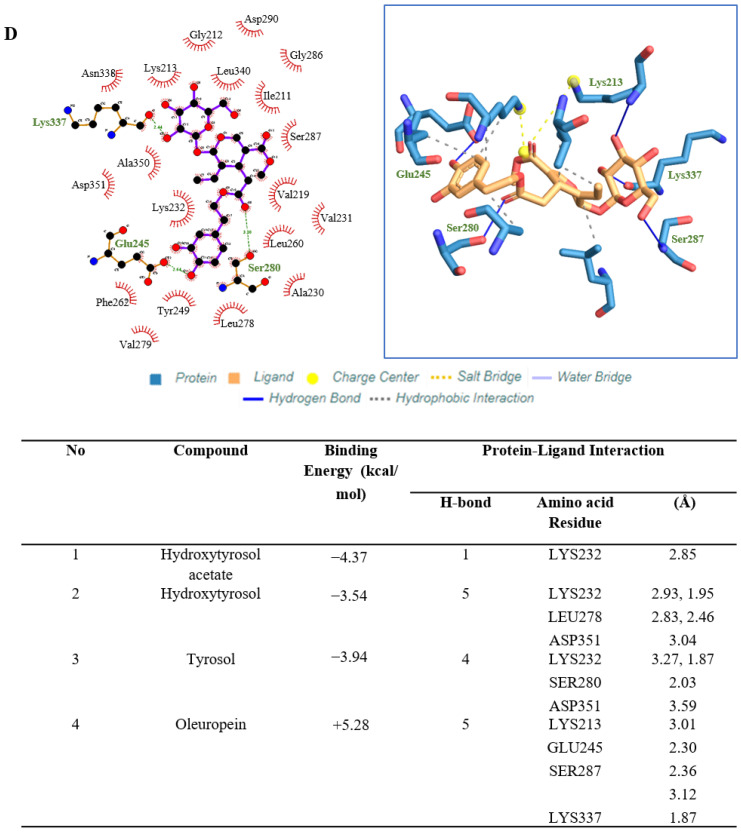
Molecular docking studies show ligand binding in the active pockets of ALK5 receptors. (**A**) HT acetate. (**B**) HT. and (**C**) Tyrosol. The dark blue line indicates the hydrogen bond, the dashed line indicates the hydrophobic interaction. Molecular docking studies show ligand binding in the active pockets of ALK5 receptors. (**D**) Oleuropein. The dark blue line indicates the hydrogen bond, the dashed line indicates the hydrophobic interaction.

Four active compounds (HT, HT acetate, tyrosol, oleuropein) were used for molecular docking analysis to study the interaction between ligands and proteins. Among the four compounds, HT acetate had the lowest binding energy value, followed by HT, tyrosol, and oleuropein. Apart from hydrogen binding, HT acetate had the most hydrophobic interactions compared to the other compounds (ILE211, VAL219, ALA230, LYS232, TYR249, LEU260, LEU278, LEU340). However, only one hydrogen bond linked HT acetate to the amino acid LYS232 (Figure 7), while three hydrophobic interactions were recorded for HT, namely on LYS232, LYS232, and LEU260. A hydrogen bond at LYS232, LEU278, and ASP351 was observed between HT and the ALK5 receptor (Figure 7). Tyrosol interacted hydrophobically at LYS232 and LEU260 and demonstrated hydrogen binding at LYS232, SER280, and ASP351 (Figure 7). Oleuropein had the highest binding energy and had five hydrogen bonds at LYS213, GLU245, SER280, SER287, and LYS337, which bound it to the protein ALK5. In addition, there was a hydrophobic interaction between oleuropein and ALK5 at VAL219, ALA230, LYS232, LEU278, and LEU340 (Figure 7).

## 3. Discussion

The olive is a well-known fruit with health beneficial properties, such as anti-inflammatory and antioxidant functions [31]. These favorable attributes are associated with the presence of phenolic compounds, such as oleuropein, oleocanthal, HT, and tyrosol, in the olive fruit [32,33,34].

Features similar to EMT events were reported in inflammatory diseases such as CRS [14,35,36]. Persistent injury, consistent inflammation, and signaling factors, such as TGFβ activation, are among the reasons for the occurrence for tissue remodeling processes. Chronic inflammation, such as in CRS, is generally accompanied by tissue remodeling, which is important to restore the structural and physiological function of damaged tissue during healing. However, prolonged inflammation can cause pathological changes, such as extracellular matrix (ECM) deposition, goblet cell hyperplasia, subepithelial edema, epithelial layer shedding, and basement membrane thickening, which can occasionally lead to nasal polyps [8,37].

Another adverse outcome of tissue remodeling is fibrosis. Typically, during normal physiological repair, myofibroblasts aid the secretion of ECM proteins and healing, and undergo apoptosis when re-epithelization is complete. However, prolonged myofibroblast activity can cause fibrosis. EMT may contribute to the existence of myofibroblasts during fibrosis. In a mouse model study, Kim et al. [38] demonstrated that RECs differentiated into fibroblasts and myofibroblasts during EMT and caused fibrosis.

These transitions can be investigated by observing the changes in epithelial and mesenchymal protein markers. The expression levels of epithelial protein markers, such as E-cadherin and ZO-1, are decreased in cells undergoing EMT. E-cadherin and ZO-1 are among the important components of the epithelial adherens junction. Reductions in these protein markers suggested that epithelial cells had begun to detach from one another and migrate [39]. The expression levels of mesenchymal protein markers, such as vimentin and αSMA, increase as cells begin to change to a mesenchymal phenotype.

Previously, we observed the potential of *O. europaea* extracts in preventing TGFβ1-induced EMT in human nasal RECs [32]. In the present study, we examined the potential of a *O. europaea* phenolic compound, i.e., HT, to modulate TGFβ1-induced EMT in human RECs. We also explored approaches in HT supplementation on EMT in TGFβ1-induced human RECs.

Among the *O. europaea* phenolic compounds, HT modulates the EMT signaling pathway. HT is produced from the hydrolysis of oleuropein, which takes place during fruit maturation, fruit processing, and the ingestion of olive fruits [40]. The potential therapeutic effect of HT was covered extensively in a review by Hu et al. [23], who reported that HT has anti-cancer, cardioprotective, and neuroprotective potential in addition to being a natural antioxidant.

EMT can be divided into type 1 (occurs during development), type 2 (occurs during wound healing and fibrosis), and type 3 (occurs during cancer progression) [6]. Although the EMT outcome can be pathologically different, e.g., cancer vs. fibrosis, a common set of pathways enables EMT. HT decreased the expression levels of the WNT co-receptors LRP6, β-catenin, SNAIL, and SLUG and increased E-cadherin expression in a breast cancer cell line [30]. Furthermore, HT reduced AKT and ERK expression levels in skin cancer, colon cancer, and hepatocellular carcinoma cells [30,41]. WNT, AKT, and ERK are proteins involved in EMT. However, only a few studies to date have investigated the effect of HT on EMT diseases other than cancer. Natural products, such as wheatgrass and quercetin, can modulate type 2 EMT diseases, such as renal fibrosis and lung fibrosis [17,42], when similar pathways are targeted. Therefore, in the present study, we induced normal human RECs with TGFβ1 to mimic the EMT conditions that occur during rhinosinusitis.

Experiments on other cell types demonstrated that HT modulated EMT according to the dose and treatment approach. Nevertheless, there has been no published study on the cytotoxic effects of HT on RECs. However, the data demonstrated clearly that high HT concentrations cause cytotoxic effects. Han et al. reported that 12-h treatment with 50 μg/mL HT (324 μM) induced apoptosis in MCF-7 cells [28]. Warletta et al. reported no significant changes in growth in cells that had been treated for 24 h with 100 μM HT (15.4 μg/mL) [43], where REC viability began to decrease at 10 μg/mL HT, thereby indicating that the RECs underwent cell death at high HT concentrations. Cell growth is important for wound healing mechanisms and tissue repair. Higher concentration of HT might cause cell death; meanwhile, exposure to a low concentration of HT might not be effective in giving the intended effect. Optimal HT concentration need to be chosen to proceed with the study. Therefore, we used 0.025, 1, 10, 15, 30, and 50 μg/mL HT for the long-term growth kinetics study. HT did not exert long-term effects on RECs at concentrations < 10 μg/mL, where REC proliferation increased significantly from day 0 until day 5. However, the total cell numbers of the RECs treated with 15, 30, and 50 μg/mL HT did not increase significantly after day 1. This result was also observed in the REC proliferation rate, which decreased and was significantly lower than that in the control.

Next, we examined EMT activation in RECs via the expression of protein markers after HT treatment, where the experiment was performed with 1 and 10 μg/mL HT. These concentrations were chosen based on the slight increase in the REC proliferation rate following treatment with 1 μg/mL HT as compared with the control, and 10 μg/mL HT was selected as it represented the initial point of gradual decrease in cell proliferation. The cell viability and proliferation were not significantly different between both concentrations. However, the vimentin and E-cadherin expression of the treated cells differed between treatment approaches, suggesting that HT potentially modulates the EMT event.

In this study, six conditions were used to mimic the possible conditions during an EMT event. Group H+T emulated the event in which HT was administered simultaneously with the EMT event. TGF pre-induction mimicked the event in which EMT occurred first, followed by HT treatment. For HT pre-treatment, HT supplementation was administered throughout the period, and EMT occurred during that time.

The effects of HT treatment and the duration of exposure to these active compounds in vitro were not widely reported. Olive leaf extract (OLE), olive oil, and HT exerted protective effects on cells and animal models [44,45,46,47,48,49,50,51]. These findings demonstrated that OE and its active compounds exert a protective effect on cells if administered before the insult occurs. The effects of using natural products in pre-treatment of EMTs were widely reported [45,52,53,54]. Therefore, we examined the effects of HT treatment methods in this study.

EMT is a dynamic and reversible event. Hence, most cells generally undergo partial EMT, in which one cell can express mesenchymal (vimentin) and epithelial (E-cadherin) phenotypes. Vimentin and E-cadherin are the downstream markers of several EMT pathways, such as the SMAD, AKT and MAPK pathways [55].

In this study, TGFβ1-induced RECs demonstrated a larger surface area, larger cell perimeter, and a higher migration rate compared to untreated RECs. Furthermore, the TGFβ1-induced RECs exhibited higher vimentin and SNAIL/SLUG expression but lower E-cadherin expression. Additionally, TGFβ1 caused AKT and SMAD2/3 phosphorylation. AKT signaling pathway activation is the reason that the RECs in the TGF group were larger than the untreated RECs. Our findings were in line with those reported by previous studies, where TGFβ1 induction activated the SMAD2/3 and AKT signaling pathways in cells [56,57,58].

The effect of HT on the TGFβ1-induced RECs was clearly observed in the H+T groups, where the addition of HT aided REC size and shape retention. Additionally, HT also reduced the cell migration rate, reduced vimentin protein expression, and decreased pAKT and pSMAD2/3 expression. Decreased pSMAD2 and pSMAD3 protein expression indicated the inhibition of TGFβ1-induced EMT [6]. Lupinacci et al. reported that OLE inhibited the migration of Met5A cells with TGFβ1-induced EMT [59]. In these cells, OLE treatment successfully decreased the fibrogenic and EMT expression markers (αSMA, N-cadherin, vimentin, fibronectin), where reduced fibrogenic expression led to reduced cell motility and migration. Moreover, the authors demonstrated the ability of OLE to reduce SNAIL, pSMAD3, and pSMAD4 protein expression. OLE contains similar phenolic compounds to the extract of olive fruits, i.e., oleuropein and HT.

We also observed the effect of HT on cells that had undergone EMT. First, the RECs were induced using TGFβ1 for 48 h before HT treatment. However, HT could not restore the original REC morphology and did not impede cell migration or decrease the expression levels of the protein markers vimentin, pAKT, and pSMAD. The findings indicated that HT cannot aid the reduction of the effects of EMT in cells that have undergone EMT.

The pre-treatment effects of HT before EMTs are also studied. RECs were found to maintain cell size similar to normal RECs and stop cell migration well compared to all groups studied. In addition, TGFβ1-induced RECs treated using HT before induction were found to maintain E-cadherin marker expression, and lower expression of SNAIL/SNUG protein markers, vimentin, pAKT and pSMAD. This suggests that HT has the ability to protect cells from EMTs.

The protective effect of natural compounds in preventing or reducing the effects of EMT has been reported previously [52,53,54,60,61]. During EMT activation, ligand binding to TGFβ receptor causing formation of SMAD complex. During EMT activation, ligand binding to TGFβ receptor causes SMAD complex formation. The complex will eventually bind to the SNAIL1 promoter and activate SNAIL transcription, which will lead to suppression of the gene related to E-cadherin [6]. In TGFβ1-induced cells, curcumin pre-treatment inhibited SNAIL expression by inhibiting SMAD2 phosphorylation and inhibiting SNAIL nuclear translocation and suppressing its transcription [52]. Wang et al. (2019) hypothesized that amygdalin (an active compound from bitter almonds) may compete for TGFβ1 receptors, thus affecting SMAD2 and SMAD3 phosphorylation [62].

Therefore, we examined the effect of ligand binding on TGFβ1 receptor type 1 or ALK5 using in silico molecular docking. Binding energy is generated when a molecule interacts with its target, where a lower binding energy value indicates more stable attachment of the complex to the target. Several small molecules, such as LY2109761, galunisertib (LY2157299), LY364947, and SB505124, attach to ALK5 and affect its activity. ALK5 inhibitors specifically replace ATP adhesion to the ALK5 kinase domain that would originally phosphorylate SMAD2 and SMAD3. The ALK5 blockade will cause SMAD signaling pathway blockade [63]. In this study, we have reported the binding energies of hydroxytyrosol acetate (−4.37 kcal/mol), hydroxytyrosol (−3.54 kcal/mol), tyrosol (−394 kcal/mol) and oleuropein (+5.28 kcal/mol). It can be seen that HT acetate has the lowest value followed by hydroxytyrosol and tyrosol. This indicates that HT has the potential to bind to ALK5 while inhibiting the phosphorylation activity of the protein associated with it. However, when compared to known ALK5 inhibitors, such as LY364947 (−10.2 kcal/mol), SD-208 (−10.0 kcal/mol) and SB505124 (−10.2 kcal/mol) the binding energy for the tested compound is indeed lower compared to the aforementioned ALK5 inhibitors [63].

Although our understanding of the effect of HT on cells in which EMT has been induced remains incomplete, HT can nevertheless modulate an EMT event. To determine the effect of HT on EMT marker expression, the effect of the experimental conditions of our study on protein and gene expression levels should be investigated to assess the EMT status. The effect of HT on the EMT pathway should be investigated by considering the activation of specific upstream EMT markers, such as SMAD, AKT, and ERK, to determine the exact effect of HT on EMT. Furthermore, to understand HT modulation of cell survival and proliferation, the activation of downstream EMT markers, especially in the AKT pathway, e.g., GSK-3β, should be investigated.

## 4. Materials and Methods

This study was approved by the Universiti Kebangsaan Malaysia Research Ethics Committee (FF-2017-363). All methods and experiments were performed in accordance with the relevant guidelines and regulations issued by the committee.

Redundant human nasal turbinate tissues were obtained with written consent from four Asian patients who had undergone turbinectomy. The turbinate tissue was washed with Dulbecco’s phosphate-buffered saline (DPBS; Gibco, NY, USA) to remove blood and mucus. Next, the epithelial layer was separated from the tissue and minced before being digested in 0.6% collagenase type I (Worthington, Lakewood, NJ, USA) for 60 min in a shaker incubator at 37 °C. After complete tissue digestion, the sample was centrifuged for 5 min at 2370× *g*. Then, the supernatant was discarded, the pellet washed with DPBS, and recentrifuged for 5 min at 2370× *g*.

The cell pellet was suspended in growth medium consisting of airway epithelial growth medium (PromoCell, Heidelberg, Germany), defined keratinocyte serum-free medium (Gibco, NY, USA) and Dulbecco’s modified Eagle’s medium:Nutrient Mixture F-12 supplemented with 5% fetal bovine serum (BioWest, Nuaillé, France) in a 1:1:2 ratio and seeded into a 6-well plate (Thermo Fisher Scientific, Waltham, MA, USA). The cells were cultured at 37 °C in 5% CO_2_ in an incubator.

The medium was changed every 2 days until the cells were 80–90% confluent. Subsequently, the REC and fibroblast co-culture was differentially trypsinized to remove fibroblasts from the culture plate. The medium was changed every 2 days until the cells were 80–90% confluent before being trypsinized in passages 1 (P1) and 2 (P2), which were used in subsequent experiments.

### 4.1. Cytotoxicity Assay

We purchased 3-Hydroxytyrosol (HT) (catalog no. H4291) from Sigma-Aldrich (St. Louis, MO, USA). The cytotoxic effect of HT was evaluated using a Vybrant™ 3-(4,5-dimethylthiazol-2-yl)-2,5-diphenyltetrazolium bromide (MTT) cell proliferation assay kit (Invitrogen, Waltham, MA, USA) following the manufacturer’s recommendations. The effect of short-term HT exposure on RECs was studied by growing P1 RECs in a 48-well plate and treating them for 24 h with 0.025–200 μg/mL HT. One untreated group was used as the control. Subsequently, medium from the HT-treated groups and untreated group was removed and replaced with 200 μL fresh basal medium. Three wells containing basal medium only were prepared as the blank/medium control. A total of 20 μL of MTT solution (final concentration, 0.5 mg/mL) were added to the wells, incubated for 4 h at 37 °C before 200 μL of 10% sodium dodecyl sulphate–HCl (SDS-HCl) solution was added and incubated further for another 4 h. The solution in the wells was divided into two replicates, and 210 μL solution was transferred into one well of a 96-well plate before the absorbance was read at 570 nm. Several optimum concentrations (0.05, 0.1, 0.2, 0.5, 1, 15, 30, and 50 μg/mL) were chosen to be used for further experimentation.

### 4.2. Quantification of Total Cells Attached and Cell Proliferation

The treated and untreated RECs were observed for 5 days to determine the long-term effect on cell growth in the HT-treated RECs. At day 0, images of five independent fields were captured randomly. These initial points will be saved in the NIS-Elements integrated Nikon microscope software. The same point will be used to capture the same image field until day 5. The total number of cells attached to the surface and the proliferation rate were calculated and quantitated using the following equation:

$$Total\;cells\;attached=\frac{Average\;cell\;count}{Objective\;area\;of\;the\;microscope}$$

$$Proliferation\;rate\;{\left(h\right)}^{-1}=\frac{ln\left[\;Total\;cells\;attached\;\left(final\right)÷Total\;cell\;attached\;\left(initial\right)\right]}{Time}$$

### 4.3. HT Supplementation and TGFβ1 Induction

**Figure 8:**
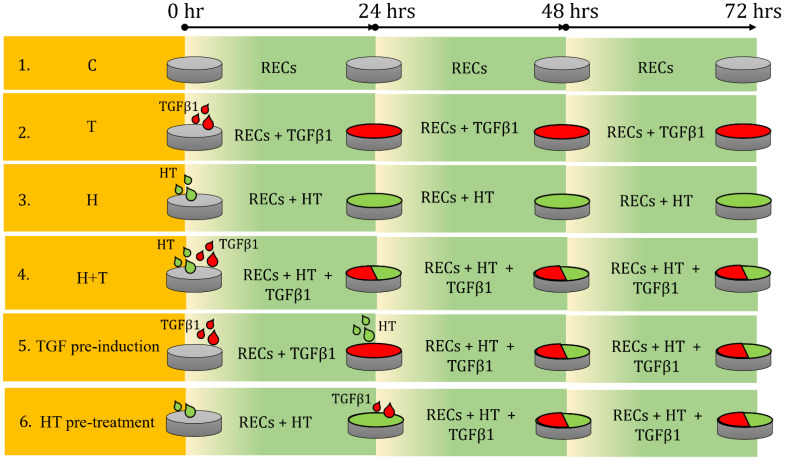
Illustration of experimental design of HT treatment of RECs. Six groups of HT and TGFβ1 treatment conditions are shown. C = Control (untreated RECs), T = TGFβ1, and H = hydroxytyrosol.

RECs at approximately 40% confluency were seeded and tested in six conditions (Figure 8): (1) untreated (C); (2) TGFβ1-supplemented (T); (3) HT-supplemented (H); (4) 72-h simultaneous supplementation of TGFβ1 and HT (H+T); (5) addition of HT after 24-h TGFβ1 supplementation and culturing in the presence of both factors for another 48-h (TGF pre-induction); (6) addition of TGFβ1 after 24-h HT supplementation and cultured in the presence of both factors for another 48 h (HT pre-treatment). The TGFβ1 concentration used in this study was 10 ng/mL.

These six approaches were tested to mimic possible conditions during treatment of an EMT event, i.e., an EMT event within CRS. Group H+T represented the event where the HT treatment took place simultaneously with the EMT event. TGF pre-induction mimicked the event in which EMT or disease, i.e., CRS, occurred first and was then treated with HT. For HT pre-treatment, the HT supplementation was administered before the EMT event occurred and subsequently continued concurrently with the EMT event or the disease.

### 4.4. Immunocytochemical Analysis

The RECs were treated with HT and TGFβ1 (Figure 8). Then, E-cadherin and vimentin expression levels were evaluated using immunocytochemical analysis following the method of Rabiatul et al. [32]. The cells were washed with DPBS, fixed in 4% paraformaldehyde for 30 min (Sigma-Aldrich, USA), permeabilized for 20 min with 0.5% Triton X-100 solution (Sigma-Aldrich), then blocked with 10% goat serum for 1 h at 37 °C. Subsequently, the cells were incubated with 1:200 mouse anti-E-cadherin antibody (ab1416) and 1:200 rabbit vimentin antibody (ab92547; Abcam, Cambridge, UK) overnight at 4 °C. The following day, the cells were washed before being incubated with 1:300 diluted Alexa Fluor 594 anti-rabbit IgG (Invitrogen, Waltham, MA, USA) and Alexa Fluor 488 anti-mouse (Invitrogen, Waltham, MA, USA) for 1 h at 37 °C. The nuclei were counterstained with 4′, 6-diamidino-2-phenylindole (DAPI). Fluorescence images were captured with an ECLIPSE Ti fluorescence microscope (Nikon, Tokyo, Japan). The total number of cells expressing E-cadherin and vimentin was calculated from the images of five randomly selected independent fields following the equation described earlier. The images were analyzed using NIS-Elements integrated Nikon microscope software.

### 4.5. Cell Morphology Analysis

The morphological changes of all groups were evaluated through cell circularity, elongation, cell surface, and cell perimeter analyses (Figure 8). REC images from all groups were captured in five random independent fields and analyzed using ImageJ v1.53. The images were processed using the Smooth and Sharpen options before being analyzed. The parameters were selected in Analyze > Set Measurement. Then, the cell shape was traced using Freehand Selection. Next, the parameters were measured and calculated with CTRL + M. The results obtained from the process were cell surface area, cell perimeter, cell roundness, Feret, and mean Feret. Cell elongation (aspect ratio) was calculated as follows: Feret × MinFeret.

### 4.6. Cell Migration Analysis

RECs that were 100% confluent were washed with DPBS before new medium was placed in the culture container. Scratches were made in the REC monolayer using a 100-µL pipette tip. Subsequently, TGFβ1 or HT was added according to the experimental requirements. The culture container was placed in the imaging system directly, where the device took pictures every 20 min at a predetermined point for 48 h. Then, the value was used to derive the migration rate and percentage of scratch closure.

### 4.7. Western Blot Analysis

The RECs were seeded into 6-well plates and treated as described earlier. The cells were lysed in radioimmunoprecipitation assay buffer containing protease inhibitors and phosphatase inhibitors. Proteins were separated by 10% SDS–polyacrylamide gel electrophoresis and transferred onto nitrocellulose membranes, which were blocked with 5% skim milk. The blots were incubated with primary antibodies against E-cadherin (1:1000; ab1416; Abcam), vimentin (1:1000; ab92547; Abcam), AKT (MAB2055; R&D Systems, MN, USA), phosphorylated (p)AKT (1:1000; ab38449; Abcam), SMAD2/3 (AF3797; R&D Systems), pSMAD2/3 (1:1000; ab63399; Abcam), SNAIL/SLUG (1:1000; ab180714; Abcam), and β-tubulin (1:2000; ab15568; Abcam). The blots were incubated with horseradish peroxidase-conjugated secondary antibodies [anti-mouse (1:10,000; Abcam) and anti-rabbit (1:10,000; Sigma-Aldrich)] and visualized using an enhanced chemiluminescent system through gel documentation with an Amersham Imager 600. In addition to conventional Western blotting, automated protein separation and immunoblotting of the Western blots were performed using a Jess system (ProteinSimple, Bio-Techne, MN, USA). The proteins were normalized following the manufacturer’s instructions to normalize sample loading variability.

### 4.8. Molecular Docking

#### 4.8.1. Ligand Preparation

AutoDock 1.5.6 was used for docking to investigate ligand binding to macromolecules (receptors). The HT, HT acetate, tyrosol, and oleuropein 3D structures were obtained from the PubChem database. Then, their structural files were converted to GDP using Open Babel. Next, the PDB file was converted to PDBQT using AutoDock.

#### 4.8.2. Receptor Provision

Based on previous studies, the ALK5 crystallization structure (PDB ID: 1RW8, resolution: 2.4 Å) was selected from the RCSB Protein Data Bank (PDB) database. Prior to docking, heteroatoms and water molecules were removed from the ALK5 structure. Then, polar hydrogen and Kollman charges were added using AutoDock. Next, the structure was saved as a PDBQT file.

#### 4.8.3. Molecular Docking

The ALK5 grid box was determined using AutoGrid according to previous studies. The grid box parameters are as follows: grid box sizes, 16, 16, and 16; X, Y, and Z coordinates, 3.753, 15.918, and 9.96; grid point spacing, 1.0 Å. Ten confirmation modes and their respective binding forces were generated using AutoDock. The lowest binding energy yield was used for further analysis. The ALK5 protein complexes and ligands were observed using protein–ligand interaction profiler. The 2D docking for hydrogen bonding analysis and hydrophobic interactions was produced using LigPlot 1.4.5.

### 4.9. Statistical Analysis

The experiments were performed in triplicate and repeated on at least three biological samples (n = 3). Data are presented as the mean ± SEM. For statistical analysis, one-way and two-way ANOVA was used depending on the variables involved in the comparison. Tukey multiple comparison was performed, to assess the statistical significance. Statistical significance was assessed with Tukey’s multiple comparison. The statistical analysis was performed using Prism 7 (GraphPad Software Inc., San Diego, CA, USA). The results were considered statistically significant at *p* < 0.05.

## 5. Conclusions

HT can modulate EMT by maintaining the epithelial phenotype. However, additional studies using in vivo disease models are needed to confirm the utility of HT as an alternative treatment for airway diseases.

## Data Availability

The data presented in this study are available upon request from the corresponding author.

## Supplementary Materials

The supporting information can be downloaded at: https://www.mdpi.com/article/10.3390/ijms24043974/s1.
