# Supplementary material for: Approaches in Hydroxytyrosol Supplementation on Epithelial—Mesenchymal Transition in TGFβ1-Induced Human Respiratory Epithelial Cells
Source: Int J Mol Sci. 2023 Feb 16;24(4):3974. doi: 10.3390/ijms24043974 (PMC9967984; doi:10.3390/ijms24043974)
Supplement: Supplementary file 1 [file ijms-24-03974-s001.zip › ijms-2121745-supplementary-final/Supplementary Data (P-value).pdf]

Supplementary Data (P-value)

Table S1. The table shows extracted p-values for Figure 1. Dose-dependent effect of HT on RECs. RECs were treated with various concentrations of HT for 24 h, and cell viability was analysed via MTT assay. *P<0.05: significantly different from control (0 ug/ml).

HT concentration in ug/ml	P Value	Significant?
**0 vs. 0.025**	>0.9999	No
**0 vs. 0.05**	0.9954	No
**0 vs. 0.75**	0.6546	No
**0 vs. 0.1**	0.8944	No
**0 vs. 0.15**	>0.9999	No
**0 vs. 0.2**	0.9959	No
**0 vs. 0.3**	>0.9999	No
**0 vs. 0.6**	0.7831	No
**0 vs. 1**	0.5301	No
**0 vs. 2**	0.2079	No
**0 vs. 5**	0.5699	No
**0 vs. 10**	0.0005	Yes
**0 vs. 50**	<0.0001	Yes
**0 vs. 100**	<0.0001	Yes
**0 vs. 200**	<0.0001	Yes

Table S2. The table shows extracted p-values for Figure 2. Long-term effect of HT supplementation on RECs. A) Total number of cells attached vs. HT concentration. B). Proliferation rate. *P<0.05: significantly different from control (0 ug/ml).

A) Total number of cells attached vs. HT concentration.

Day	HT concentration in ug/ml	P Value	Significant?
**Day 1**	0 vs. 0.025	0.9942	No
	0 vs. 1	>0.9999	No
	0 vs. 10	0.9993	No
	0 vs. 15	0.9773	No
	0 vs. 30	0.9745	No
	0 vs. 50	0.8500	No
**Day 2**	0 vs. 0.025	0.8747	No
	0 vs. 1	0.9421	No
	0 vs. 10	0.6328	No
	0 vs. 15	0.1501	No
	0 vs. 30	0.0236	Yes
	0 vs. 50	0.0011	Yes
**Day 3**	0 vs. 0.025	0.6592	No
	0 vs. 1	0.2669	No
	0 vs. 10	0.9854	No
	0 vs. 15	0.0457	Yes
	0 vs. 30	0.0006	Yes
	0 vs. 50	<0.0001	Yes
**Day 4**	0 vs. 0.025	0.0142	Yes
	0 vs. 1	0.0052	Yes
	0 vs. 10	0.8602	No

	0 vs. 15	<0.0001	Yes
	0 vs. 30	<0.0001	Yes
	0 vs. 50	<0.0001	Yes
**Day 5**	0 vs. 0.025	0.0158	Yes
	0 vs. 1	0.0042	Yes
	0 vs. 10	0.8433	No
	0 vs. 15	<0.0001	Yes
	0 vs. 30	<0.0001	Yes
	0 vs. 50	<0.0001	Yes

B) Proliferation rate

**HT concentration in ug/ml**	P Value	Significant?
**0 vs. 0.025**	>0.9999	No
**0 vs. 1**	0.9679	No
**0 vs. 10**	0.9999	No
**0 vs. 15**	0.3061	No
**0 vs. 30**	<0.0001	Yes
**0 vs. 50**	<0.0001	Yes

Table S3. The table shows extracted p-values for Figure 3. Protein expression levels of RECs that have been treated with TGFβ1 and HT with six different approaches. A) Coexpression of E-cadherin and vimentin expression levels (%) vs control. B) E-cadherin expression (%) vs group. C) Vimentin expression (%) vs group. *P<0.05: significantly different from control (0 ug/ml).

A) Coexpression of E-cadherin and vimentin expression levels (%) vs control

**[HT]**	Experimental group	P Value	Significant?
**1 µg/mL**	CTRL vs. HT	0.6420	No
	CTRL vs. H+T	>0.9999	No
	CTRL vs. TGF pre induction	0.9061	No
	CTRL vs. HT pre treatment	>0.9999	No
	TGFB1 vs. HT	No	No
	TGFB1 vs. H+T	No	No
	TGFB1 vs. TGF pre induction	No	No
	TGFB1 vs. HT pre treatment	No	No
**10 µg/mL**	CTRL vs. HT	0.9497	No
	CTRL vs. H+T	>0.9999	No
	CTRL vs. TGF pre induction	>0.9999	No
	CTRL vs. HT pre treatment	>0.9999	No
	TGFB1 vs. HT	0.9587	No
	TGFB1 vs. H+T	0.5384	No
	TGFB1 vs. TGF pre induction	0.0354	Yes
	TGFB1 vs. HT pre treatment	0.0713	No

B) E-cadherin expression (%) vs group.

**Day**	Experimental group	P Value	Significant?

**1 µg/mL**	CTRL vs. HT	<0.0001	Yes
	CTRL vs. H+T	0.0388	Yes
	CTRL vs. TGF pre induction	0.9789	No
	CTRL vs. HT pre treatment	0.0411	Yes
	TGFB1 vs. HT	<0.0001	Yes
	TGFB1 vs. H+T	0.0338	Yes
	TGFB1 vs. TGF pre induction	0.9708	No
	TGFB1 vs. HT pre treatment	0.0359	Yes
**10 µg/mL**	CTRL vs. HT	<0.0001	Yes
	CTRL vs. H+T	0.3264	No
	CTRL vs. TGF pre induction	>0.9999	No
	CTRL vs. HT pre treatment	0.7927	No
	TGFB1 vs. HT	<0.0001	Yes
	TGFB1 vs. H+T	0.2967	No
	TGFB1 vs. TGF pre induction	>0.9999	No
	TGFB1 vs. HT pre treatment	0.7597	No

C) Vimentin expression (%) vs group.

Day	HT concentration in ug/ml	P Value	Significant?
**1 µg/mL**	CTRL vs. HT	<0.0001	Yes
	CTRL vs. H+T	0.7301	No
	CTRL vs. TGF pre induction	<0.0001	Yes
	CTRL vs. HT pre treatment	0.0302	Yes
	TGFB1 vs. HT	<0.0001	Yes
	TGFB1 vs. H+T	<0.0001	Yes
	TGFB1 vs. TGF pre induction	<0.0001	Yes
	TGFB1 vs. HT pre treatment	<0.0001	Yes
**10 µg/mL**	CTRL vs. HT	0.0841	No
	CTRL vs. H+T	0.3141	No
	CTRL vs. TGF pre induction	0.9397	No
	CTRL vs. HT pre treatment	>0.9999	No
	TGFB1 vs. HT	<0.0001	Yes
	TGFB1 vs. H+T	<0.0001	Yes
	TGFB1 vs. TGF pre induction	<0.0001	Yes
	TGFB1 vs. HT pre treatment	<0.0001	Yes

Table S4. The table shows extracted p-values for Figure 4. B) Cell circularity, C) Cell elongation, and E) Cell perimeter. *P<0.05: significantly different from control (RECs). # P<0.05: significantly different from group T.

B) Cell circularity

Experimental group	P Value	Significant?
**RECS vs. HT**	0.6201	No
**RECS vs. TGF**	0.0002	Yes
**RECS vs. HT+TGF**	0.0067	Yes
**RECS vs. TGF pre induction**	0.0109	Yes
**RECS vs. HT pre treatment**	0.0304	Yes
**HT vs. TGF**	0.0127	Yes
**HT vs. HT+TGF**	0.2298	No

HT vs. TGF pre induction	0.3156	No
HT vs. HT pre treatment	0.5550	No
TGF vs. HT+TGF	0.7703	No
TGF vs. TGF pre induction	0.6579	No
TGF vs. HT pre treatment	0.4032	No

D) Cell surface area

Experimental group	P Value	Significant?
RECS vs. HT	0.2137	No
RECS vs. TGF	0.0010	Yes
RECS vs. HT+TGF	0.5787	No
RECS vs. TGF pre induction	0.3563	No
RECS vs. HT pre treatment	0.1512	No
HT vs. TGF	0.0033	Yes
HT vs. HT+TGF	0.9774	No
HT vs. TGF pre induction	0.6142	No
HT vs. HT pre treatment	>0.9999	No
TGF vs. HT+TGF	0.2229	No
TGF vs. TGF pre induction	0.9996	No
TGF vs. HT pre treatment	0.0158	Yes

E) Cell perimeter

Experimental group	P Value	Significant?
RECS vs. HT	0.1046	No
RECS vs. TGF	<0.0001	Yes
RECS vs. HT+TGF	0.1159	No
RECS vs. TGF pre induction	0.0474	Yes
RECS vs. HT pre treatment	0.0023	Yes
HT vs. TGF	0.0001	Yes
HT vs. HT+TGF	0.6085	No
HT vs. TGF pre induction	0.1359	No
HT vs. HT pre treatment	0.2028	No
TGF vs. HT+TGF	0.0726	No
TGF vs. TGF pre induction	0.9455	No
TGF vs. HT pre treatment	0.0041	Yes

Table S5. The table shows extracted p-values for Figure 5. B) migration rate, C) closure percentage, *P<0.05: significantly different from control (RECs). # P<0.05: significantly different from group T.

B) Migration rate

Experimental group	P Value	Significant?
RECS vs. HT	0.0414	Yes
RECS vs. TGF	0.6048	No
RECS vs. HT+TGF	0.0021	Yes
RECS vs. TGF pre induction	0.9573	No
RECS vs. HT pre treatment	0.0004	Yes
HT vs. TGF	0.4695	No
HT vs. HT+TGF	0.4948	No

HT vs. TGF pre induction	0.1586	No
HT vs. HT pre treatment	0.1227	No
TGF vs. HT+TGF	0.0298	Yes
TGF vs. TGF pre induction	0.9637	No
TGF vs. HT pre treatment	0.0056	Yes

C) Closure percentage

Experimental group	P Value	Significant?
RECS vs. HT	0.0091	Yes
RECS vs. TGF	0.4956	No
RECS vs. HT+TGF	0.0163	Yes
RECS vs. TGF pre induction	0.1066	No
RECS vs. HT pre treatment	0.0002	Yes
HT vs. TGF	0.1801	No
HT vs. HT+TGF	0.9991	No
HT vs. TGF pre induction	0.6859	No
HT vs. HT pre treatment	0.2288	No
TGF vs. HT+TGF	0.2985	No
TGF vs. TGF pre induction	0.8758	No
TGF vs. HT pre treatment	0.0034	Yes

Table S6. The table shows extracted p-values for Figure 6. Densitometry analysis for tested protein markers observing the effects of HT addition and TGFβ1 influence on RECs *P<0.05: significantly different from control. # P<0.05: significantly different from group T

B) E-cadherin

Experimental group	P Value	Significant?
RECS vs. HT	0.0086	Yes
RECS vs. TGF	<0.0001	Yes
RECS vs. HT+TGF	<0.0001	Yes
RECS vs. TGF pre induction	0.0023	Yes
RECS vs. HT pre treatment	0.0108	Yes
HT vs. TGF	0.0020	Yes
HT vs. HT+TGF	0.0108	Yes
HT vs. TGF pre induction	0.9624	No
HT vs. HT pre treatment	>0.9999	No
TGF vs. HT+TGF	0.8938	No
TGF vs. TGF pre induction	0.0072	Yes
TGF vs. HT pre treatment	0.0016	Yes

C) Vimentin

Experimental group	P Value	Significant?
RECS vs. HT	0.9811	No
RECS vs. TGF	0.8360	No
RECS vs. HT+TGF	0.3147	No
RECS vs. TGF pre induction	0.3352	No
RECS vs. HT pre treatment	0.1187	No
HT vs. TGF	0.4661	No
HT vs. HT+TGF	0.6718	No

HT vs. TGF pre induction	0.1235	No
HT vs. HT pre treatment	0.3244	No
TGF vs. HT+TGF	0.0498	Yes
TGF vs. TGF pre induction	0.9267	No
TGF vs. HT pre treatment	0.0165	Yes

C) Snail-Slug

Experimental group	P Value	Significant?
RECS vs. HT	0.9954	No
RECS vs. TGF	0.9154	No
RECS vs. HT+TGF	0.9175	No
RECS vs. TGF pre induction	0.9772	No
RECS vs. HT pre treatment	0.9998	No
HT vs. TGF	0.9958	No
HT vs. HT+TGF	0.9961	No
HT vs. TGF pre induction	>0.9999	No
HT vs. HT pre treatment	0.9718	No
TGF vs. HT+TGF	>0.9999	No
TGF vs. TGF pre induction	0.9997	No
TGF vs. HT pre treatment	0.8141	No

F) pAKT

Experimental group	P Value	Significant?
RECS vs. HT	0.9983	No
RECS vs. TGF	0.0188	Yes
RECS vs. HT+TGF	0.5549	No
RECS vs. TGF pre induction	>0.9999	No
RECS vs. HT pre treatment	0.7614	No
HT vs. TGF	0.0066	Yes
HT vs. HT+TGF	0.8002	No
HT vs. TGF pre induction	0.9933	No
HT vs. HT pre treatment	0.9366	No
TGF vs. HT+TGF	0.0002	Yes
TGF vs. TGF pre induction	0.0263	Yes
TGF vs. HT pre treatment	0.0005	Yes

G) pSMAD2

Experimental group	P Value	Significant?
RECS vs. HT	0.8657	No
RECS vs. TGF	0.0014	Yes
RECS vs. HT+TGF	0.8887	No
RECS vs. TGF pre induction	0.0151	Yes
RECS vs. HT pre treatment	0.9820	No
HT vs. TGF	0.0086	Yes
HT vs. HT+TGF	>0.9999	No
HT vs. TGF pre induction	0.0975	No
HT vs. HT pre treatment	0.9972	No

**TGF vs. HT+TGF**	0.0078	Yes
**TGF vs. TGF pre induction**	0.7000	No
**TGF vs. HT pre treatment**	0.0041	Yes

H) pSMAD3

Experimental group	P Value	Significant?
**RECS vs. HT**	0.3767	No
**RECS vs. TGF**	0.0006	Yes
**RECS vs. HT+TGF**	0.0028	Yes
**RECS vs. TGF pre induction**	0.0003	Yes
**RECS vs. HT pre treatment**	0.0968	No
**HT vs. TGF**	0.0149	Yes
**HT vs. HT+TGF**	0.0831	No
**HT vs. TGF pre induction**	0.0067	Yes
**HT vs. HT pre treatment**	0.9339	No
**TGF vs. HT+TGF**	0.9017	No
**TGF vs. TGF pre induction**	0.9959	No
**TGF vs. HT pre treatment**	0.0702	No
